# Is the muscle–tendon architecture of non-athletic Kenyans different from that of Japanese and French males?

**DOI:** 10.1186/s40101-023-00326-3

**Published:** 2023-06-01

**Authors:** Yoko Kunimasa, Kanae Sano, Caroline Nicol, Joëlle Barthèlemy, Masaki Ishikawa

**Affiliations:** 1 Graduate School of Sport and Exercise Sciences, Osaka University of Health and Sport Sciences, Kumatori-Cho, Sennan-Gun, Osaka, 590-0496 Japan; 2 ISM, CNRS & Aix-Marseille University, 13288 Marseille, France; 3 Faculty of Education, Niigata University, Niigata, Niigata 950-2181 Japan; 4 Faculty of Engineering, Niigata University, Niigata, Niigata 950-2181 Japan; 5 Faculty of Health and Well-being, Kansai University, Sakai, Osaka 590-8515 Japan

**Keywords:** Muscle-tendon unit, Achilles tendon, Muscle architecture, Ultrasonography, Kenyans

## Abstract

**Background:**

In endurance running, elite Kenyan runners are characterized by longer thigh, shank, and Achilles tendon (AT) lengths combined with shorter fascicles and larger medial gastrocnemius (MG) pennation angles than elite Japanese runners. These muscle-tendon characteristics may contribute to the running performance of Kenyans. Furthermore, these specific lower-leg musculoskeletal architectures have been confirmed not only in elite Kenyan runners but also in non-athletic Kenyans since early childhood. However, it remains questionable whether the differences in muscle-tendon architecture between Kenyans and Japanese differ from those of European Caucasians. Therefore, this study aimed to compare anthropometry and muscle–tendon architecture of young non-athletic Kenyan males with their Japanese and French counterparts.

**Methods:**

A total of 235 young non-athletic males, aged 17–22 years, volunteered. The anthropometric measures, thigh, and shank lengths, as well as AT and MG muscle architecture, were measured using ultrasonography and a tape measure. Inter-group differences in anthropometry and muscle-tendon architecture were tested using one-way ANOVA and ANCOVA analyses controlling for shank length and muscle thickness.

**Results:**

The anthropometric and muscle-tendon characteristics of the non-athletic French were closer to those of the Kenyans than to those of the Japanese. However, the ultrasonography analysis confirmed that the non-athletic Kenyans had the longest AT as well as the shortest MG fascicles and the largest pennation angle compared to the French and Japanese, even after controlling for shank length and muscle thickness with ANCOVA, respectively.

**Conclusions:**

These results confirmed the specificity of the muscle-tendon architecture of the triceps surae in Kenyans in comparison to their Japanese and French counterparts in non-athletic adults. This study provides additional support to the fact that Kenyans may have musculotendinous advantages in endurance running.

## Background

In bipedal locomotion, musculoskeletal architecture and its mechanical behavior are crucial for economical movements and power production [1]. Several studies reported that inherent advantages of the lower-limb architecture can affect athletic performance [2–5]. In endurance running, the specific skeletal and muscle-tendon architecture of lower-leg muscles of the Kenyan runners may contribute to their running performance [6, 7], such as a long Achilles tendon (AT) as well as a thin medial gastrocnemius (MG) muscle with short fascicle length and large pennation angle. These specific lower-leg muscle-tendon architecture has been confirmed not only in elite Kenyan runners but also in non-athletic Kenyans compared to Japanese [8]. Furthermore, this study found that the decrease in MG fascicle length and increase pennation angle observed for the adult Japanese with the increase in running performance level resulted in a lack of difference between elite Kenyans and Japanese. These findings suggested that the unique muscle-tendon architecture of the Kenyan population may be genetically determined and contribute to the dominance of Kenyans in middle- and long-distance races. However, to further test this hypothesis, it would be interesting to investigate whether the muscle-tendon architecture of the triceps surae is characteristic of Kenyans compared to other non-Japanese populations, even after controlling for anthropometric differences.

Previous anthropometric studies have reported that Kenyan endurance runners and young western Kenyans are characterized by long and light lower legs compared to their Danish counterparts [9, 10]. As the AT length is approximately proportional to the tibia bone length and body height [11], it was expected that the long AT length of Kenyans may still hold even when compared to other European Caucasians than the Danes. Since body mass index reference values of Danes aged 0–45 years best match the French reference values, especially for men [12], we thought it would be interesting to compare the anthropometry and muscle-tendon architecture of young Kenyan non-athletes with those of the Japanese population and the Caucasian French population. We hypothesized that similar to the elite Kenyan runners, the non-athletic Kenyans would have longer AT and thinner MG muscles with shorter fascicle length and larger pennation angle compared to not only the Japanese but also the French. This hypothesis could hold even after controlling for the shank length and muscle thickness with ANCOVA.

## Methods

A total of 235 young healthy males (age range 17–22), 54 Kenyans, 81 French Caucasian, and 100 Japanese volunteered (Table 1). Japanese were recruited and tested in different cities (i.e., Osaka, Hyogo, Shiga, Okayama, Hiroshima, and Fukuoka) between 2012 and 2015. Kenyans were recruited and tested in Nairobi in March 2015 and March 2016 or Kitui (160 km away) in February 2016. French were recruited and tested in Gap (in the south of France) in September 2014. This study was performed according to the guidelines of the Declaration of Helsinki and was approved by the local Ethics Committee of the Osaka University of Health and Sport Sciences (approval number: 16–1).

**Table 1:** Anthropometric parameters for Kenyans, French, and Japanese

	**Kenyan (*n* = 54)**	**French (*n* = 81)**	**Japanese (*n* = 100)**
Age (years)	18.5 ± 1.3	18.4 ± 1.3	18.5 ± 1.3
Body height (cm)	169.1 ± 5.6	177.5 ± 6.5***	171.6 ± 5.2*_†††_
Body mass (kg)	58.6 ± 6.1	67.8 ± 8.4***	60.6 ± 6.1†††
Thigh length (cm)	40.1 ± 1.7	39.3 ± 2.0*	38.7 ± 2.5***
Thigh length (% height)	23.8 ± 0.9	22.2 ± 0.9***	22.6 ± 1.2***†
Shank length (cm)	39.0 ± 1.6	37.6 ± 2.1***	35.4 ± 1.9***†††
Shank length (% height)	23.1 ± 0.7	21.2 ± 0.7***	20.6 ± 0.7***†††

Group mean ± SD values

^*^ and *** significant difference between Kenyan vs. French and Japanese (*p* < 0.05 and *p* < 0.001, respectively)

^†^ and ††† significant difference between French vs. Japanese (*p* < 0.05 and *p* < 0.001, respectively)

Body mass was measured by electric scale (RD-503, Tanita Corp., Japan). Body height, thigh, and shank lengths were measured using tape measure and carpenter square [6]. AT and MG muscle architecture was measured using ultrasonography [Noblus with a 55-mm linear array probe (model: L55, 5–13 MHz), Hitachi Ltd., Japan] [6, 8]. All parameters were measured bilaterally while standing at rest and averaged for both legs. Thigh length was defined as the distance from the greater trochanter to the proximal head of fibula and shank length from the outer femoral condyle to the tip of the lateral malleolus. AT length was quantified using ultrasonography and tape measures from the point of its distal insertion into the calcaneus to the AT junction between medial and lateral gastrocnemii muscles [8].

MG fascicle length, its pennation angle, and muscle thickness were measured from midsagittal ultrasound images of the MG muscle belly. The length of one fascicle located in the center of the MG ultrasound image was measured as the distance between its insertions into the superficial and deep aponeuroses [13] and digitized using analysis software (ImageJ, NIH, Bethesda, MD, USA). The muscle thickness was the largest perpendicular distance between deep and superficial aponeuroses. The MG pennation angle was measured between the MG fascicle insertion points in the superficial and deep aponeuroses and another point on the deep aponeurosis. Inter-day repeatability of the muscle architecture measured by the same experimenter was confirmed [8].

Statistical analysis was performed using IBM SPSS Statistics (version 27.0, IBM Corp. Armmonk, NY, USA). Values in the text are reported as mean ± SD. Inter-group differences for each parameter were tested by one-way ANOVA analyses. An additional one-way ANCOVA was conducted to determine statistically significant differences between Kenyans, Japanese, and French on the AT length controlling for the shank length and on the MG pennation angle and fascicle length controlling for the MG muscle thickness, respectively. Further post hoc analyses were conducted with a Bonferroni adjustment. We used an alpha level of 0.05 for all statistical tests.

## Results

Group means and statistical comparisons of anthropometry of Kenyan, French, and Japanese are shown in Table 1. The body mass of the Kenyans and Japanese was lighter than that of French. The body height was shorter in the Kenyans than in the French and Japanese. The absolute and relative (% body height) values of thigh and shank lengths were significantly longer in the Kenyans than in their French and Japanese counterparts, respectively. In the comparison between French and Japanese groups, the relative thigh length as well as the absolute and relative shank lengths was longer in the French.

**Fig. 1:**
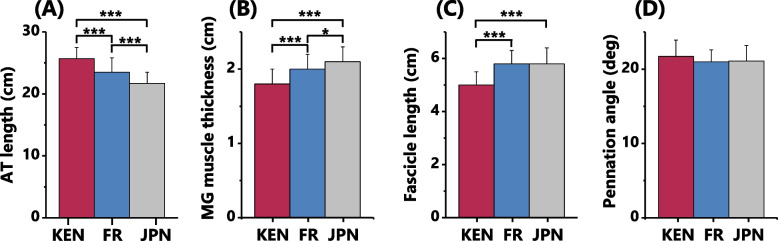
Comparison of muscle-tendon architecture between Kenyans (KEN), French (FR), and Japanese (JPN). Results are shown as mean ± SD. Inter-group differences for each parameter were tested by one-way ANOVA analyses. *** and * indicate significant differences (*p* < 0.001 and *p* < 0.05, respectively)

**Fig. 2:**
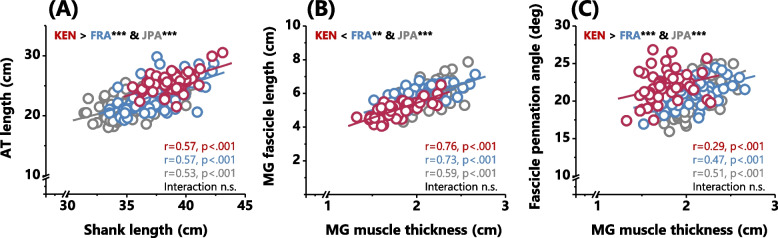
Achilles tendon (AT) and medial gastrocnemius (MG) muscle architecture of Kenyan (KEN), French (FR), and Japanese (JPN) adult males. One-way ANCOVA was conducted on the AT length controlling for the shank length (**A**) and on the MG fascicle length and pennation angle controlling for the MG muscle thickness (**B** and **C**, respectively). *** and ** indicate significant inter-group differences (*p* < 0.001 and *p* < 0.01, respectively)

Regarding muscle-tendon architecture, the Kenyans had longer AT (Fig. 1A) and smaller MG muscle thickness (Fig. 1B) than the French and Japanese, respectively. The relative AT length to the shank length was 65.9 ± 3.7, 62.6 ± 5.1, and 61.4 ± 4.3% for the Kenyans, French, and Japanese, respectively. The ANCOVA analyses confirmed that there was a significant effect of group on the AT length after controlling for the shank length [F(2, 232) = 15.567, *p* < 0.01]. Post hoc tests showed significantly longer AT in Kenyans than in Japanese and French (*p* < 0.001, respectively) (Fig. 2A). The MG fascicle length was shorter in the Kenyans than in the French and Japanese in both absolute (Fig. 1C) and relative scales (12.9 ± 1.4 vs. 15.5 ± 1.6 and 16.5 ± 2.0%, *p* < 0.001 for both comparisons). The ANCOVA analyses confirmed a significant group effect on fascicle length and pennation angle after controlling for the MG muscle thickness [fascicle length: F(2232) = 11.668, *p* < 0.001, pennation angle: F(2232) = 11.129, *p* < 0.001]. Post hoc tests showed that Kenyans had significantly shorter MG fascicle length than Japanese and French (*p* < 0.01 and *p* < 0.001, respectively) and larger pennation angle (*p* < 0.001, respectively) than Japanese and French (Fig. 2B, C). In the comparison between French and Japanese groups, the French had longer absolute AT (Fig. 1A), smaller MG muscle thickness (Fig. 1B), and relatively shorter MG fascicle length (*p* < 0.001).

## Discussion

The anthropometric and muscle-tendon characteristics of the French were closer to those of the Kenyans than those of the Japanese. However, the ultrasonography analysis confirmed that the non-athletic Kenyans had the longest AT as well as the shortest MG fascicle length and the largest pennation angle compared to the French and Japanese, even after controlling for shank length and muscle thickness with ANCOVA.

The dominance of Kenyans in endurance running was thought to be due to genetic factors, as first suggested by Bengt Saltin’s group [9, 10, 14, 15]. Not only elite Kenyan runners but also Kenyan boys have better running economy than their Danish counterparts, which is explained by their thinner calf muscles [10]. Consistent with this finding, our previous study showed that Kenyan runners and non-athletes, including boys, had a thinner MG muscle with a longer AT and shorter MG fascicles compared to Japanese [8]. The relationships between these unique structural and functional characteristics suggest that Kenyans have an inherent muscle-tendon advantage for endurance running [6–8]. The present results provide additional support for the hypothesis that Kenyans have some unique muscle-tendon characteristics, compared not only to the Japanese but also to the French population. Interestingly, this uniqueness remained even after controlling for anthropometric differences between Kenyans, French, and Japanese. In contrast to the greater contribution of MG fascicle pennation angle to greater MG muscle thickness in European Caucasians [16, 17], Kenyans had the shortest MG fascicle length and the greatest pennation angle, although their MG muscle thickness was the lowest. These results bring additional support to the genetic origin of the unique muscle-tendon architecture of Kenyans [8].

The results of this study add to the existing literature on the intrinsic musculoskeletal architecture of the lower leg in Kenyans, including comparisons with different populations, but some limitations deserve to be mentioned. First, contrasting with our previous comparison between Kenyans and Japanese males [8], the current data were collected from a limited number of people and a limited age range of 17–22. In addition, the current study did not include elite runners, although Kenyan’s muscle-tendon architecture may be considered a prerequisite for becoming elite runners in endurance running events. While Kenyans would have a specific muscle-tendon architecture by the age of 8 years old [8], the current age range of 17–22 is after the growth period. Finally, the comparisons were limited to three male populations, with only the Japanese group included participants from different cities and locations. Future studies should include elite runners and investigate the relationship between muscle-tendon architecture, running cost, and performance.

## Conclusion

This study provides additional information consistent with the specifics of the muscle-tendon architecture of the triceps surae in Kenyans by comparing them to Japanese and French non-athletic young adults. Regardless of shank length, Kenyans had the longest AT, and regardless of MG muscle thickness, the shortest muscle fascicles and the largest pennation angle. These results suggest again that Kenyans can possess inherent muscle-tendon advantages for endurance running. Such muscle-tendon architecture may be considered as a prerequisite for becoming elite runners in endurance running events. Further research should include data from elite endurance runners and provide a more comprehensive picture of the relationships between the structural lower-leg characteristics and the running cost and performance.

## Data Availability

The data in this study are available from the corresponding author upon reasonable request.

## Abbreviations

AT: Achilles tendon
MG: Medial gastrocnemius
